# Fear of COVID-19 Among Patients with Inflammatory Bowel Disease as Compared to Patients with Other Gastrointestinal Conditions

**DOI:** 10.5152/tjg.2022.21774

**Published:** 2022-08-01

**Authors:** Bobby Lo, Manuel Barreiro-de Acosta, Charles N. Bernstein, Johan Burisch, Nuno Ferreira, Richard B. Gearry, Antonina Mikocka-Walus, Anna Mokrowiecka, Inês A. Trindade, Simon R. Knowles

**Affiliations:** 1 Department of Gastroenterology, Copenhagen University Hospital, Hvidovre, Denmark; 2 Unit of Inflammatory Bowel Disease, University Hospital of Santiago de Compostela, Spain; 3 Department of Medicine Rady Faculty of Health Sciences, University of Manitoba, Winnipeg, MB, Canada; 4 University of Manitoba IBD Clinical and Research Centre, Winnipeg, MB, Canada; 5 Department of Social Sciences, University of Nicosia, Cyprus; 6 Department of Medicine, University of Otago, Christchurch, New Zealand; 7 Deakin University Faculty of Psychology, Geelong, Melbourne, Australia; 8 Department of Digestive Tract Diseases, Medical University of Lodz, Poland; 9 CINEICC, University of Coimbra Faculty of Psychology and Education Sciences, Portugal; 10 Department of Molecular and Clinical Medicine, Institute of Medicine, Sahlgrenska Academy, University of Gothenburg, Sweden; 11 Department of Psychological Sciences, Swinburne University of Technology, Melbourne, Australia

**Keywords:** COVID-19, fear, inflammatory bowel disease

## Abstract

**Background:**

Although several studies have reported the impact of fears relating to coronavirus-19 on several chronic illnesses, there are few studies focused on gastrointestinal conditions. Therefore, the aim of this study was to compare the fear of coronavirus-19 in patients with inflammatory bowel disease to other gastrointestinal conditions and how the fear of COVID-19 manifests across different demographical backgrounds among inflammatory bowel disease respondents.

**Methods:**

Participants with gastrointestinal conditions (age ≥ 18) were recruited from 27 countries. Demographic, clinical, and psychosocial information was collected. An adapted scale for inflammatory bowel disease patients measuring the fear of coronavirus-19 and gastrointestinal-specific fear of coronavirus-19 was used.

**Results:**

In 831 participants (312 inflammatory bowel disease), only significant increases in gastrointestinal–fear of coronavirus-19 were found in between inflammatory bowel disease and other gastrointestinal conditions (mean [standard deviation]: 13.5 [5.5] vs 10.9 [5.0],* P* < .01). Among inflammatory bowel disease respondents, persons on sick leave had significantly more fear of coronavirus-19 than those employed (median [IQR], 31.0 [28.5-39.5] vs 26.0 [20.0-33.0], *P* = .035) and significantly more gastrointestinal–fear of coronavirus-19 compared to the employed (18.0 [14.5-22.0] vs 13.0 [9.0-17.0],* P* = .033) or respondents outside of the labor market (12.0 [7.0-16.0], *P* = .022). Persons living in a rural setting had significantly more fear of coronavirus-19 compared to persons living in regional setting (29.5 [22.0-37.8] vs 25.0 [20.0-31.3], *P* = .007) and gastrointestinal–fear of coronavirus-19 (15.0 [11.0-19.8] vs 12.0 [9.0-16.0], *P* = .02).

**Conclusion:**

Respondents with inflammatory bowel disease are more afraid of coronavirus-19 regarding their disease; especially, persons on sick leave or persons living in a rural setting. This should be taken into consideration to personalize the support that health care providers can offer in mitigating fear related to coronavirus-19.

**Main Points**

- Inflammatory bowel disease (IBD) patients have significantly more fear of COVID-19 (FoC) in regards to their (gastrointestinal) GI disease than patients with other GI diseases.
- Patients with IBD living in rural areas experience significantly more general and GI-specific FoC (GI-FoC) compared to those living in metropolitan or regional areas.
- Patients with IBD who are on sick leave have a significantly more general and GI-FoC than those employed and those employed or outside the labor market, respectively.
- During consultations in this and future pandemics, physicians should have more focus on IBD patients in regards to handling their fear and concerns, especially those living in rural areas and/or on sick leave.

## Introduction

Coronavirus-19 (COVID-19), a disease caused by a new severe acute respiratory syndrome virus believed to originate in the Wuhan region of China, was declared a pandemic on March 12, 2020.^1^ As of September 2021, more than 218 million infections have been registered, with over 4.5 million deaths and death rates that continue to rise daily.^2^

In response to the pandemic, a range of measures have been taken in an attempt to control the spread of the virus, including partial or complete community shutdowns and mandatory self-isolation and quarantines.^3^ While these measures are found to be helpful in suppressing the pandemic, these can also contribute to increased fear of contamination and death.^4^ In addition, studies have shown that mental health has been adversely affected by the COVID-19 measures in up to 50% of the populations.^5,6^

Chronic gastrointestinal (GI) diseases, such as inflammatory bowel disease (IBD), are associated with high rates of comorbid anxiety and depression independent of the pandemic.^7^ This makes these populations at risk for further mental health deterioration. However, few studies have investigated the COVID-19-related fears experienced by patients with GI diseases.^8,9^ In IBD, specifically in its 2 main subtypes Crohn’s disease (CD) and ulcerative colitis (UC), anxiety and depression have been frequently reported due in part to the disease burden and the side effects of its treatments regardless of the disease activity.^7,10^ In addition, anxiety and depression can be exacerbated by the added stress posed by the virus’ potentially lethal risks, particularly when the additional risk posed by using immunomodulatory medications was initially unknown.^11^

For those living with IBD, navigating the immense quantities of COVID-19 information available can be challenging, potentially leading to doubt, fear, or unnecessary self-isolation.^12,13^ The advice offered by different organizations as to whether IBD patients are at increased risk of contracting COVID-19 and/or the likelihood of experiencing severe complications if infected^14-16^ was inconsistent and often contradictory.

The fear of COVID-19 (FoC) and its association with other psychological and health outcomes among patients with IBD have not been fully explored.^9,17^ However, given a high psychological burden of GI conditions in general,^18^ it is interesting to determine whether the fears experienced by persons with IBD differ from persons with other GI conditions in relation to COVID-19. For example, the rates of anxiety/depression in irritable bowel syndrome (IBS) are higher than in IBD,^19^ yet reliance on immunomodulating drugs might make those with IBD more fearful of the COVID-19 consequences.

Furthermore, few studies have investigated whether demographic characteristics such as employment status, education, or geographical region impact patients’ fears of COVID-19.^20^ Perhaps, the fears of COVID-19 might be tempered by having greater resource availability (e.g., education and employment) or that a person living in a region with a high infection rate will be more fearful than a person living in a region with a lower rate.^2^

The aim of this study was to compare how FoC manifests in patients with IBD compared to other GI conditions and how FoC manifests across different demographical backgrounds (employment status, education level, and country of residence) among IBD respondents using a large international sample.

## Materials and Methods

### Design

This is a cross-sectional study, nested within an ongoing cohort, using a web-based questionnaire (administrated by Qualtrics) to assess the wellbeing of patients with GI diseases during the COVID-19 pandemic. The study design has been described elsewhere.^21^ The study was advertised across multiple countries via GI-specific patient organizations and associated social media.

The survey was administered in English only. The completion time was approximately 40 minutes.

### Participants

All participants were above 18 years of age with a self-reported GI condition diagnosed by a doctor (e.g., IBD, IBS, and eosinophilic esophagitis) and were recruited from 27 countries.

The questionnaire was administrated by Qualtrics. By completing the questionnaire, the participants consented to participate in the study. The study was approved by the Swinburne University of Technology Human Research Ethics Committee (Ref: 20202978-4430).

### Measures

Demographic data included age, gender, marital status, dependents, cohabitors, geographical region, residential setting, educational level, and employment status.

Information regarding IBD-related drugs included the use of 5-aminosalicylic acid, corticosteroids, immunomodulators, and biologics. In addition, informations regarding their GI condition in the past month and the number of comorbidities were included.

**Supplementary Figure 1.**
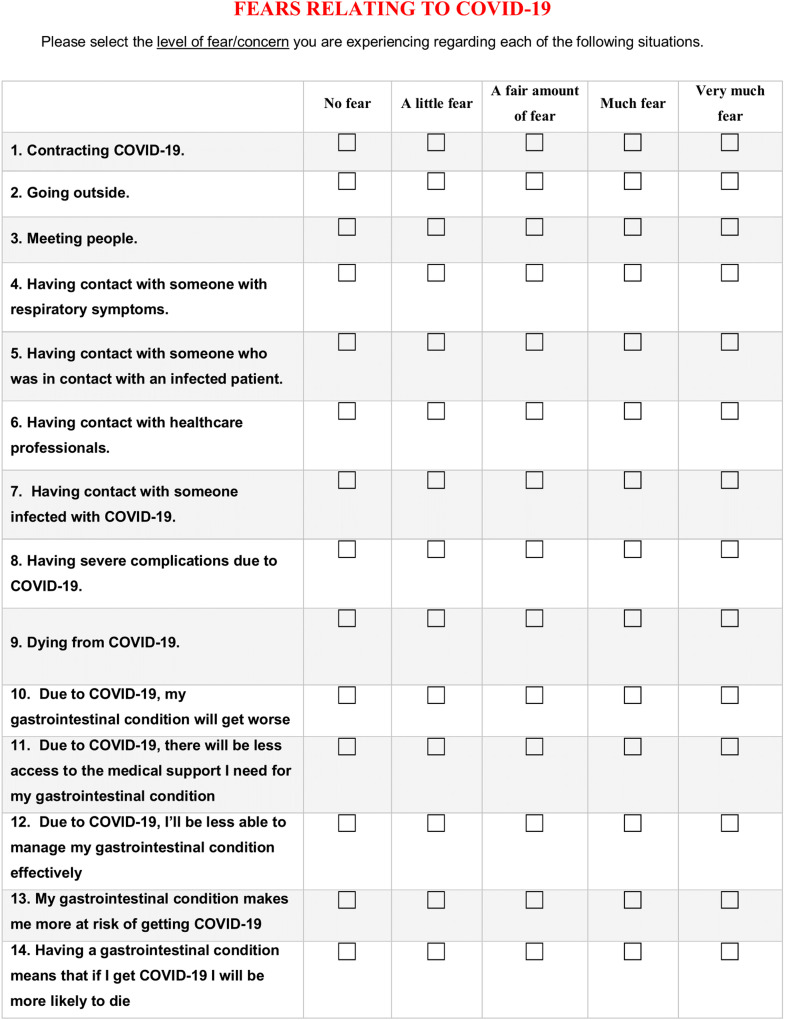
Fears relating to COVID-19 questionnaire.

The Fear Relating to COVID-19 Scale is an adaptation of the scale developed by Trindade and Ferreira (2020)^9^ measuring FoC (original items 1-9) and validated in IBD. For this study, 5 GI-specific FoC questions were added (GI-FoC; new items 10-14, see Supplementary Figure 1). Thus, the scale has 14 items that ask respondents to indicate their level of fear/concern regarding different situations (e.g., contracting COVID, having contact with health professionals). The questionnaire was completed on a Likert scale of 1-5, ranging from “No fear” to “Very much fear.” A higher score indicates more fear or greater concern about COVID-19. The scale presents general FoC and GI-FoC, both with acceptable internal reliability (α = 0.93 and α = 0.88, respectively).

### Statistical Analysis

As appropriate, data are presented as mean and standard deviation (SD) or median and interquartile range (IQR). As appropriate, comparisons between groups were carried out using Student’s *t*-test, Wilcoxon signed-rank test, one-way analysis of variance, or *χ* ^2^ test. All data were analyzed using R (R Core Team [2019], R: A language and environment for statistical computing, R Foundation for Statistical Computing, Vienna, Austria. URL https://www.R-project.org/).

Patients with IBD, and those with other GI conditions, were compared to the general FoC and GI-FoC. Analyses of subgroups of persons with IBD were carried out according to their educational level, geographical region, and employment status. Sensitivity analysis was carried out on an item-specific level for both the general FoC and GI-FoC.

Linear regression models were developed for the dependent variables of FoC and GI-FoC, with age, gender, marital status, dependents, cohabitors, geographical region, educational level, employment status, and medical treatments as independent variables. The best-fitting models were selected using the Akaike information criterion (AIC). Results from the linear regression models are reported as estimated coefficients at the 95% CI.

For analytical purposes:

- Gender was divided into male, female, and other.
- Marital status was divided into single status (singles, widowed, divorced, and separated) and with a partner (married and de facto).
- The question of cohabitation was divided into living alone or with others.
- The geographical regions were divided into Asia, Africa, North America, South America, Europe, and Australia and New Zealand.

Educational level was divided into low (elementary school, some high school with no diploma, and high school degree or equivalent), medium (some college credit with no degree, trade/technical/vocational training and associate degree), and high (bachelor’s degree, Master’s degree, professional degree, or doctorate).

The residential setting was divided into metropolitan, regional, and rural (rural and remote settings).

Current employment status was based on the status 2 weeks before the study. It was divided into being employed (full-time employed, part-time employed, casually employed, self-employed, or a student), unemployed, outside the labor market (retired, pensioners, and home duties), insecure employment situation (e.g., redundancy or as described by the respondents), and sick leave (disability pension, sickness benefits, or other circumstances described by the respondents).

## Results

In total, 831 persons participated, of whom 312 were IBD respondents (CD = 175 [56.1%], UC = 116 [37.2%], and IBD unidentified = 21 [6.7%]). Overall, the IBD participants were younger, more likely to be male, single, had more dependents, residents of Australia and New Zealand, full-time employed than persons with other GI conditions. Conversely, the non-IBD participants had more comorbidities compared to the IBD group. Demographic data for both IBD and the non-IBD groups are shown in Table 1.

### Fear of COVID-19 in IBD Versus Other GI Conditions

Overall, there was no significant difference, in general, in FoC between persons with IBD and persons with other GI conditions (IBD vs. non-IBD, mean [SD]: 26.80 [8.85] vs. 26.34 [8.30], t = −0.74, *df* = 622.32*, P* = .46). However, there was a significant difference in GI-FoC found between groups, with IBD participants presenting higher levels of GI-FoC (mean [SD]: 13.5 [5.5] vs. 10.9 [5.0], t = −6.97, df = 605.69, *P* < .01). The greatest fears described by participants with IBD were that their condition would worsen, that there would be a negative impact on their access to medical support, being at increased risk of contracting COVID-19, and being at increased risk of death if they contracted COVID-19 (Supplementary Table 1).

### Fear of COVID-19 by Demographic Characteristics Among IBD Patients

**Figure 1.**
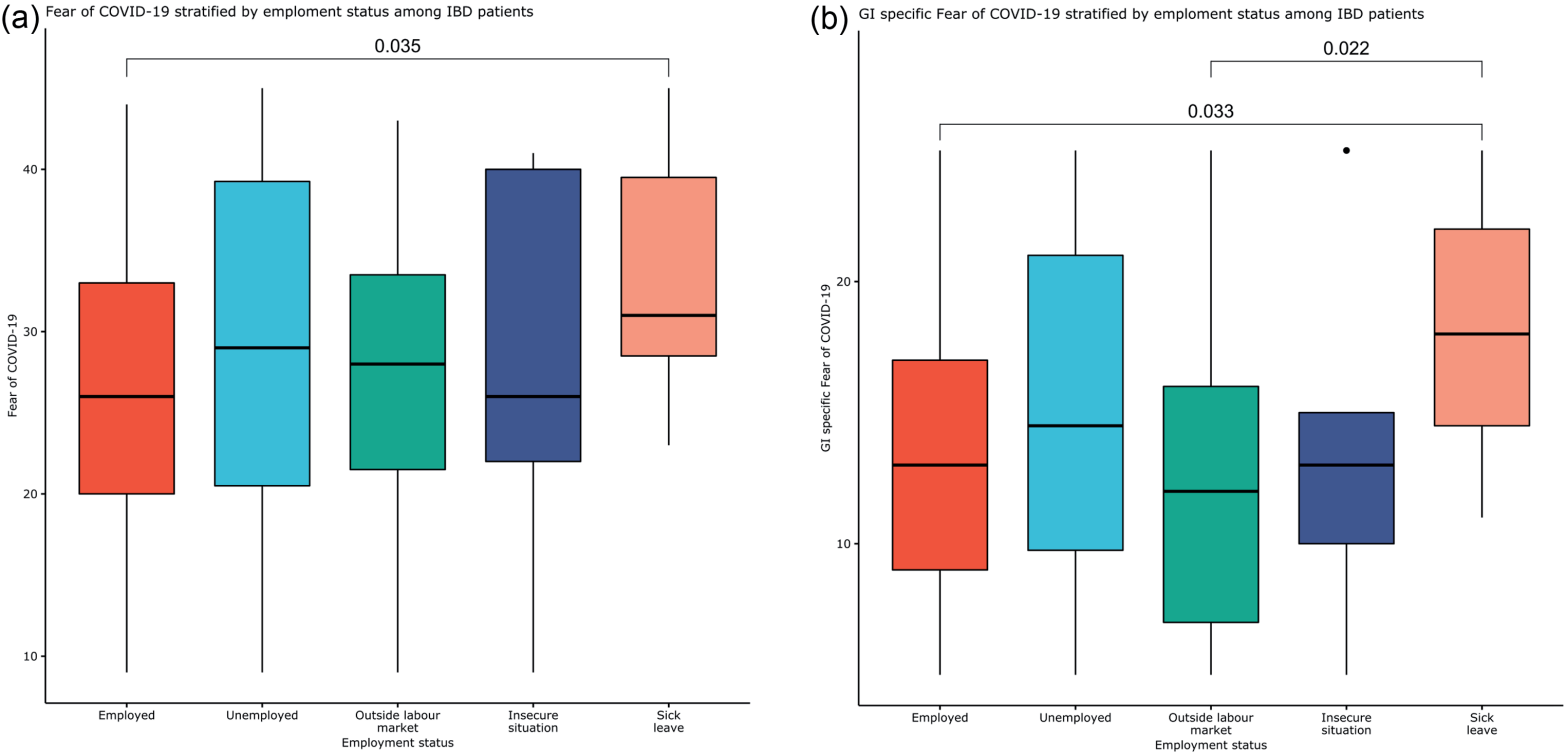
(A-B.) Fear of COVID-19 stratified by employment status among IBD patients (A) and GI-specific fear of COVID-19 stratified by employment status among IBD patients (B). IBD, inflammatory bowel disease; COVID-19, coronavirus disease 19; GI, gastrointestinal.

Among IBD respondents, there was no general trend according to employment status. However, respondents on sick leave had significantly more general FoC than those employed and significantly more GI-FoC than the employed or respondents outside the labor market (Figures 1A and B). In addition, sensitivity analysis demonstrated a significant group difference in being fearful of contracting COVID-19, having contact with someone with respiratory symptoms, having contact with healthcare professionals, and death in the event of contracting COVID-19 due to their IBD (Supplementary Table 1).

**Figure 2.**
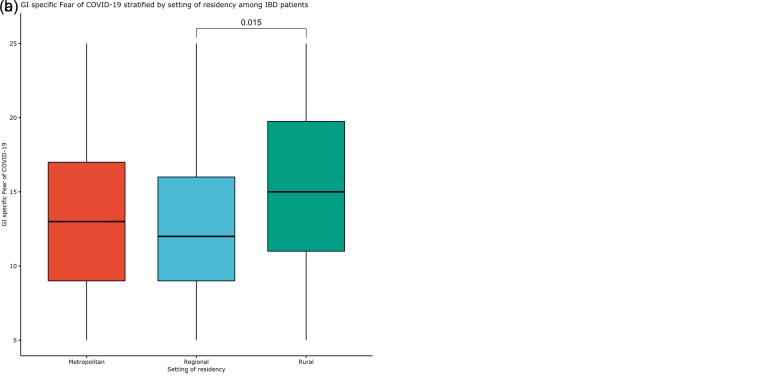
A-B. Fear of COVID-19 stratified by setting of residency among IBD patients (A) and GI-specific fear of COVID-19 stratified by setting of residency among IBD patients (B). IBD, inflammatory bowel disease; COVID-19, coronavirus disease 19; GI, gastrointestinal.

IBD participants living in a rural setting had significantly more general FoC and GI-FoC (Figures 2A and B). In addition, sensitivity analysis demonstrated a significant group difference in being fearful of contracting COVID-19, going outside, meeting people, having contact with someone who was in contact with an infected person, having contact with someone infected with COVID-19, that their IBD puts them at greater risk of contracting COVID-19, and that their IBD means that if they contract COVID-19, they will be more likely to die (Supplementary Table 1).

There were no differences in general FoC and GI-FoC according to educational background. However, in the sensitivity analysis, participants with more advanced education were less afraid of dying from COVID-19 (Supplementary Table 1).

The best fit model for general FoC included gender, age, residential setting, and employment status. We further added information on their GI condition the past month and their medical treatment to the model. This resulted in an intercept of 32.00 [95% CI: 27.80 to 36.21]; age was the only variable associated with the outcome, with −0.12 [95% CI: −0.20 to −0.04] per year, indicating the older the age, the less general FoC.

Using the same method to assess GI-FoC, the best linear regression model used gender, age, residential setting, and employment status. We further added information on their GI condition the past month and their medical treatment to the model. This resulted in an intercept of 17.62 [95% CI: 15.04 to 20.20]; age (−0.08 per year [95% CI: −0.13 to −0.03], indicating the older the age, the lower the GI-FoC score), living in a rural setting (2.23 [95% CI 95: 0.60 to 3.87]), being on sick leave (5.19 [95% CI: 0.40 to 9.97]), GI condition being rarely active (−2.68 [95% CI: −4.72 to −0.65]), and GI condition being in remission the past month (2.11 [CI 95% CI: −4.06 to −0.15]) were significantly associated with GI-FoC.

## Discussion

In this cross-continental, cross-sectional survey, we have shown that patients with IBD have higher levels of GI-FoC compared to patients with other GI diseases. General FoC was similar between these groups. Persons on sick leave and/or living in a rural setting have increased FoC.

Fear of COVID-19 has been frequently reported in the general population.^20^ An international survey, similarly designed to our study, focused on identifying factors associated with COVID-19 fear conducted in March 2020 with a healthy sample (n = 439). The study showed that health anxiety, regular media and social media use, and risks for loved ones, all accounting for nearly 40% of the variance, were significantly associated with COVID-19 fear.^20^ In another study, female sex was associated strongly with FoC.^22^ However, a recent meta-analysis^23^ (n = 33 studies) linked the FoC largely to other mental health problems such as anxiety, traumatic stress, depression, and insomnia. Since the rates of symptoms of common mental disorders have been high during the pandemic, it is unsurprising that FoC has been prevalent as well.

It is also not unexpected that persons with a GI disorder present a considerable level of both general and GI-FoC. While patients with IBD and patients with other GI disorders did not differ on their levels of general FoC, and IBD participants presented higher levels of GI-FoC. IBD respondents seem to be more afraid of worsening their condition during the pandemic, a negative impact on access to medical support, and being at increased risk of contracting COVID-19 and death due to COVID-19. This could be because patients with IBD are using immunomodulating drugs, including corticosteroids which have been associated with poorer outcomes during the coronavirus infection.^24^ However, this study’s IBD respondents’ general FoC contrasts with a previous study conducted with IBD participants from Portugal.^9^ The mentioned study was conducted during the lockdown at the first peek of the pandemic (April to May 2020) and found a considerably higher level of general FoC with the same questionnaire (mean score of 35.59 [6.21]). These results suggest that general FoC may have decreased over time among persons with IBD as the pandemic progressed and the distress of living with the restrictions imposed by the pandemic became less.

Interestingly, our study found that while IBD participants reported greater fears about having less access to medical support, they did not fear that the management of their diseases would be affected. This likely reflects the anxiety associated with having a chronic disease and experiencing COVID-19 infection but not necessarily outcomes for the chronic disease itself. On the other hand, at the beginning of the pandemic, some media information referred that IBD patients, especially those receiving immunosuppressive or biological drugs, could be at higher risk for COVID-19 increasing the FoC. This has later been debated intensely, and currently, patients receiving immunosuppressives (excl. corticosteroids) and biological drugs are not considered at a higher risk of COVID-19.

Respondents living in a rural setting reported more general FoC and GI-FoC. This may reflect their perceptions or the reality of differential access to care. It has been shown that rural areas tend to have greater challenges in access to medical care and a higher proportion of persons with no health insurance. Therefore, lack of means to seek/receive healthcare could be an additional element contributing to FoC. Further to that, the work profiles in rural areas tend to focus on jobs that cannot be done remotely (e.g., agriculture, mining, forestry, and fishing) and that in most cases are deemed essential, increasing, therefore, the possibility of exposure to the virus, hence increasing FoC.^25^ The rapid decline for some critically ill patients with COVID-19 that has been widely publicized may have heightened these concerns, especially for the expertise and resources available in rural hospitals. Furthermore, persons on sick leave experience significantly higher FoC and GI-FoC than those employed and those employed or outside the labor market, respectively. This may reflect the fact that persons with higher disabilities have a lower quality of life and that persons with low socioeconomic status do worse with their disease in general.^26^

Our study’s main strengths were that this was a multi-continental study conducted at the height of the pandemic. However, some limitations include being an English survey that may have limited responses in countries where English was not the primary language. This, paired with the fact that it took approximately 40 minutes to complete the questionnaire, limited the number of participants. While the sample size was substantial, it was modest for the stratification of groups that risk both types I and II errors. Due to the study design, responses were self-reported only. Therefore, we could not confirm the diagnosis or utilize standard disease activity measures (e.g., CD activity index) or biological markers (e.g., fecal calprotectin).

Our observations highlight several factors that should be considered by both public health officials and clinicians managing patients with IBD. First, as vaccination rollouts accelerate globally, high rates of GI-FoC among some IBD patients may lead to higher demand, including new anxieties over delays in accessing vaccine.^27^ Adverse effects from the vaccines, even if uncommon, may also exacerbate anxieties.^28^ Changes to GI-FoC and FoC following vaccination rollout will also be of interest, although they may depend on vaccine efficacy and safety in IBD patients. Within the IBD population, there is already inequity in patient-care between countries, rural, and urban places of residence and places of employment.^29^ The present study suggests that the pandemic is likely to have increased these inequities and contributed to increased fear and anxiety. To address these issues, precise and effective public health messaging is required to reach all patients with IBD, regardless of where they live. Broad public health messaging is vital, but specific messaging to patients with IBD through healthcare providers is also likely to lead to improved outcomes. Examples of clinic-led COVID-19 health messaging to IBD patients have shown to be effective.^28^ Interventions that may mitigate fear and anxiety should also be studied prospectively in order to reduce IBD patient distress and suffering. Lastly, we did not include other psychosocial perspectives in the analysis, such as anxiety, resilience, coping methods, etc., which could explain the experienced fear in further detail. However, we believe this study design represents the “true” fear even when all these factors are not adjusted for by not including these dimensions. Nonetheless, we have in the study group partly investigated the complexity of fear during COVID 19.^30^

## Conclusion

Respondents with IBD are more fearful of the consequences of COVID-19 due to their disease than other GI diseases; especially, persons on sick leave or persons living in a rural setting. These findings should be taken into consideration to personalize the support health care providers can offer in mitigating the fear related to COVID-19 among persons with IBD. In addition, new interventions to mitigate fear and anxiety among IBD patients should also be studied to reduce distress and suffering.

## Figures and Tables

**Table 1.** Demographic Information Stratified by IBD Patients and Patients with Other GI Conditions

Demographic Variables	Non-IBD		IBD	*P*
N	519		312	
Age (mean [SD])	53.11 (16.05)		42.96 (15.39)	<.001
Gender (%)				<.001
Equally/neither/unsure	3 (0.6)		0 (0.0)	
Female	450 (86.7)		234 (75.0)	
Male	65 (12.5)		77 (24.7)	
Others	1 (0.2)		1 (0.3)	
Marital status (%)				<.001
De facto	25 (4.8)		29 (9.3)	
Divorced	35 (6.7)		15 (4.8)	
Married	317 (61.1)		147 (47.1)	
Separated	9 (1.7)		3 (1.0)	
Single	117 (22.5)		115 (36.9)	
Widowed	16 (3.1)		3 (1.0)	
Living with (%)				.307
Alone	88 (17.0)		51 (16.3)	
Friends	8 (1.5)		6 (1.9)	
Other	55 (10.6)		43 (13.8)	
Parents	37 (7.1)		31 (9.9)	
Partner	331 (63.8)		181 (58.0)	
Dependents (mean [SD])	0.50 (0.97)		0.75 (1.14)	.001
Geographical region (%)				<.001
Asia	1 (0.2)		2 (0.6)	
Australia and New Zealand	67 (12.9)		103 (33.0)	
Europe	392 (75.5)		173 (55.4)	
North America	58 (11.2)		34 (10.9)	
South America	1 (0.2)		0 (0.0)	
Education highest (%)				.021
Associate degree	18 (3.5)		21 (6.7)	
Bachelor’s degree	175 (33.7)		104 (33.3)	
Doctorate degree	23 (4.4)		10 (3.2)	
Elementary school to 8th grade	6 (1.2)		1 (0.3)	
High school degree or equivalent	46 (8.9)		38 (12.2)	
Master’s degree	90 (17.3)		60 (19.2)	
Professional degree	28 (5.4)		8 (2.6)	
Some college credit, no degree	53 (10.2)		22 (7.1)	
Some high school, no diploma	19 (3.7)		20 (6.4)	
Trade/technical/vocational training	61 (11.8)		28 (9.0)	
Employment status (%)				<.001
Casually employed	7 (1.3)		7 (2.2)	
Full-time employed	146 (28.1)		124 (39.7)	
Home duties	23 (4.4)		15 (4.8)	
Others	31 (6.0)		18 (5.8)	
Part-time employed	68 (13.1)		41 (13.1)	
Pensioner	34 (6.6)		14 (4.5)	
Retired	127 (24.5)		26 (8.3)	
Self-employed	30 (5.8)		19 (6.1)	
Student	18 (3.5)		20 (6.4)	
Unemployed	35 (6.7)		28 (9.0)	
Living setting (%)				.017
Metropolitan	169 (32.6)		122 (39.1)	
Regional	192 (37.0)		124 (39.7)	
Remote	3 (0.6)		0 (0.0)	
Rural	155 (29.9)		66 (21.2)	
Symptoms past month (%)				.608
Often active, giving me symptoms most days	92 (17.7)		67 (21.5)	
Sometimes active, giving me symptoms on some days (for instance, 1-2 days/week)	90 (17.3)		43 (13.8)	
Constantly active, giving me symptoms every day	96 (18.5)		62 (19.9)	
I was well in the past month, what I consider a remission or absence of symptoms	104 (20.0)		58 (18.6)	
Occasionally active, giving me symptoms 1-2 days a fortnight	54 (10.4)		35 (11.2)	
Rarely active, giving me symptoms on a few days in the past month	83 (16.0)		47 (15.1)	
Number of comorbidities (mean [SD])	2.01 (1.10)		1.72 (1.01)	<.001
No medication (%)	164 (31.6)		102 (32.7)	.802
5-Aminosalicylates (%)	0 (0.0)		52 (16.7)	<.001
Biologics (%)	1 (0.2)		63 (20.2)	<.001
Corticosteroids (%)	9 (1.7)		22 (7.1)	<.001
Immunomodulators (%)	8 (1.5)		57 (18.3)	<.001

IBD, inflammatory bowel disease; SD, standard deviation; GI, gastrointestinal.

**Supplementary Table 1.** Fear Relyated to COVID-19

Question	non-IBD	IBD	P	Employment status						Residental setting				Education level			P
Employed	Unemployed	Outside the labour market	Insecure situation	Sick leave	P	Metropolitan	Regional	Rural	P	Low education	Medium education	High education
N	519	312		217	28	55	5	7		122	124	66		59	43	210	
Contracting covid-19 (%)			.276						.014				.007				.210
No fear	51 (9.8)	35 (11.2)		66 (30.4)	7 (25.0)	20 (36.4)	1 (20.0)	3 (42.9)		34 (27.9)	41 (33.1)	22 (33.3)		17 (28.8)	22 (51.2)	58 (27.6)	
A little fear	200 (38.5)	102 (32.7)		78 (35.9)	6 (21.4)	16 (29.1)	0 (0.0)	2 (28.6)		42 (34.4)	50 (40.3)	10 (15.2)		20 (33.9)	11 (25.6)	71 (33.8)	
A fair amount of fear	166 (32.0)	97 (31.1)		34 (15.7)	3 (10.7)	5 (9.1)	0 (0.0)	0 (0.0)		16 (13.1)	12 (9.7)	14 (21.2)		10 (16.9)	3 (7.0)	29 (13.8)	
Much fear	59 (11.4)	42 (13.5)		19 (8.8)	4 (14.3)	10 (18.2)	2 (40.0)	0 (0.0)		11 (9.0)	14 (11.3)	10 (15.2)		5 (8.5)	4 (9.3)	26 (12.4)	
Very much fear	43 (8.3)	36 (11.5)		20 (9.2)	8 (28.6)	4 (7.3)	2 (40.0)	2 (28.6)		19 (15.6)	7 (5.6)	10 (15.2)		7 (11.9)	3 (7.0)	26 (12.4)	
Going outside (%)			.886						.180				.026				.989
No fear	220 (42.4)	127 (40.7)		42 (19.4)	8 (28.6)	9 (16.4)	1 (20.0)	0 (0.0)		22 (18.0)	23 (18.5)	15 (22.7)		13 (22.0)	9 (20.9)	38 (18.1)	
A little fear	170 (32.8)	99 (31.7)		78 (35.9)	5 (17.9)	14 (25.5)	1 (20.0)	1 (14.3)		44 (36.1)	42 (33.9)	13 (19.7)		16 (27.1)	14 (32.6)	69 (32.9)	
A fair amount of fear	93 (17.9)	60 (19.2)		9 (4.1)	4 (14.3)	3 (5.5)	1 (20.0)	1 (14.3)		7 (5.7)	2 (1.6)	9 (13.6)		4 (6.8)	3 (7.0)	11 (5.2)	
Much fear	27 (5.2)	18 (5.8)		84 (38.7)	10 (35.7)	27 (49.1)	2 (40.0)	4 (57.1)		46 (37.7)	55 (44.4)	26 (39.4)		25 (42.4)	16 (37.2)	86 (41.0)	
Very much fear	9 (1.7)	8 (2.6)		4 (1.8)	1 (3.6)	2 (3.6)	0 (0.0)	1 (14.3)		3 (2.5)	2 (1.6)	3 (4.5)		1 (1.7)	1 (2.3)	6 (2.9)	
Meeting people (%)			.489						.344				.001				.248
No fear	81 (15.6)	50 (16.0)		54 (24.9)	6 (21.4)	16 (29.1)	0 (0.0)	2 (28.6)		23 (18.9)	33 (26.6)	22 (33.3)		14 (23.7)	17 (39.5)	47 (22.4)	
A little fear	233 (44.9)	124 (39.7)		92 (42.4)	10 (35.7)	18 (32.7)	1 (20.0)	3 (42.9)		58 (47.5)	55 (44.4)	11 (16.7)		26 (44.1)	10 (23.3)	88 (41.9)	
A fair amount of fear	127 (24.5)	78 (25.0)		25 (11.5)	6 (21.4)	6 (10.9)	2 (40.0)	1 (14.3)		17 (13.9)	10 (8.1)	13 (19.7)		9 (15.3)	7 (16.3)	24 (11.4)	
Much fear	54 (10.4)	40 (12.8)		35 (16.1)	2 (7.1)	11 (20.0)	2 (40.0)	0 (0.0)		16 (13.1)	22 (17.7)	12 (18.2)		8 (13.6)	6 (14.0)	36 (17.1)	
Very much fear	24 (4.6)	20 (6.4)		11 (5.1)	4 (14.3)	4 (7.3)	0 (0.0)	1 (14.3)		8 (6.6)	4 (3.2)	8 (12.1)		2 (3.4)	3 (7.0)	15 (7.1)	
Having contact with someone with respiratory symptoms (%)			.429						.001				.065				.073
No fear	36 (6.9)	24 (7.7)		51 (23.5)	8 (28.6)	14 (25.5)	1 (20.0)	3 (42.9)		26 (21.3)	39 (31.5)	12 (18.2)		20 (33.9)	9 (20.9)	48 (22.9)	
A little fear	136 (26.2)	80 (25.6)		62 (28.6)	5 (17.9)	13 (23.6)	0 (0.0)	0 (0.0)		29 (23.8)	35 (28.2)	16 (24.2)		13 (22.0)	10 (23.3)	57 (27.1)	
A fair amount of fear	150 (28.9)	77 (24.7)		58 (26.7)	4 (14.3)	13 (23.6)	0 (0.0)	0 (0.0)		29 (23.8)	31 (25.0)	15 (22.7)		16 (27.1)	16 (37.2)	43 (20.5)	
Much fear	99 (19.1)	75 (24.0)		13 (6.0)	3 (10.7)	5 (9.1)	3 (60.0)	0 (0.0)		12 (9.8)	7 (5.6)	5 (7.6)		4 (6.8)	0 (0.0)	20 (9.5)	
Very much fear	98 (18.9)	56 (17.9)		33 (15.2)	8 (28.6)	10 (18.2)	1 (20.0)	4 (57.1)		26 (21.3)	12 (9.7)	18 (27.3)		6 (10.2)	8 (18.6)	42 (20.0)	
Having contact with someone who was in contact with an infected patient (%)			.554						.288				.028				.369
No fear	31 (6.0)	23 (7.4)		53 (24.4)	5 (17.9)	9 (16.4)	1 (20.0)	0 (0.0)		24 (19.7)	32 (25.8)	12 (18.2)		14 (23.7)	11 (25.6)	43 (20.5)	
A little fear	129 (24.9)	67 (21.5)		50 (23.0)	6 (21.4)	10 (18.2)	0 (0.0)	1 (14.3)		24 (19.7)	34 (27.4)	9 (13.6)		12 (20.3)	5 (11.6)	50 (23.8)	
A fair amount of fear	125 (24.1)	68 (21.8)		50 (23.0)	5 (17.9)	19 (34.5)	1 (20.0)	1 (14.3)		34 (27.9)	29 (23.4)	13 (19.7)		16 (27.1)	15 (34.9)	45 (21.4)	
Much fear	109 (21.0)	76 (24.4)		15 (6.9)	2 (7.1)	5 (9.1)	1 (20.0)	0 (0.0)		10 (8.2)	4 (3.2)	9 (13.6)		3 (5.1)	1 (2.3)	19 (9.0)	
Very much fear	125 (24.1)	78 (25.0)		49 (22.6)	10 (35.7)	12 (21.8)	2 (40.0)	5 (71.4)		30 (24.6)	25 (20.2)	23 (34.8)		14 (23.7)	11 (25.6)	53 (25.2)	
Having contact with healthcare professionals (%)			.554						<.001				.176				.273
No fear	157 (30.3)	104 (33.3)		46 (21.2)	4 (14.3)	17 (30.9)	2 (40.0)	1 (14.3)		24 (19.7)	29 (23.4)	17 (25.8)		15 (25.4)	14 (32.6)	41 (19.5)	
A little fear	195 (37.6)	106 (34.0)		84 (38.7)	6 (21.4)	15 (27.3)	0 (0.0)	1 (14.3)		41 (33.6)	42 (33.9)	23 (34.8)		20 (33.9)	18 (41.9)	68 (32.4)	
A fair amount of fear	108 (20.8)	70 (22.4)		13 (6.0)	2 (7.1)	3 (5.5)	0 (0.0)	1 (14.3)		10 (8.2)	4 (3.2)	5 (7.6)		2 (3.4)	1 (2.3)	16 (7.6)	
Much fear	42 (8.1)	19 (6.1)		72 (33.2)	10 (35.7)	19 (34.5)	2 (40.0)	1 (14.3)		43 (35.2)	46 (37.1)	15 (22.7)		20 (33.9)	8 (18.6)	76 (36.2)	
Very much fear	17 (3.3)	13 (4.2)		2 (0.9)	6 (21.4)	1 (1.8)	1 (20.0)	3 (42.9)		4 (3.3)	3 (2.4)	6 (9.1)		2 (3.4)	2 (4.7)	9 (4.3)	
Having contact with someone infected with covid-19 (%)			.137						.143				.027				.058
No fear	17 (3.3)	14 (4.5)		42 (19.4)	4 (14.3)	10 (18.2)	0 (0.0)	1 (14.3)		19 (15.6)	30 (24.2)	8 (12.1)		11 (18.6)	9 (20.9)	37 (17.6)	
A little fear	91 (17.5)	53 (17.0)		42 (19.4)	7 (25.0)	3 (5.5)	0 (0.0)	1 (14.3)		20 (16.4)	25 (20.2)	8 (12.1)		5 (8.5)	2 (4.7)	46 (21.9)	
A fair amount of fear	95 (18.3)	57 (18.3)		41 (18.9)	2 (7.1)	7 (12.7)	1 (20.0)	0 (0.0)		20 (16.4)	24 (19.4)	7 (10.6)		8 (13.6)	8 (18.6)	35 (16.7)	
Much fear	120 (23.1)	51 (16.3)		7 (3.2)	2 (7.1)	4 (7.3)	1 (20.0)	0 (0.0)		7 (5.7)	2 (1.6)	5 (7.6)		2 (3.4)	1 (2.3)	11 (5.2)	
Very much fear	196 (37.8)	137 (43.9)		85 (39.2)	13 (46.4)	31 (56.4)	3 (60.0)	5 (71.4)		56 (45.9)	43 (34.7)	38 (57.6)		33 (55.9)	23 (53.5)	81 (38.6)	
Having severe complications due to covid-19 (%)			.250						.442				.380				.466
No fear	31 (6.0)	22 (7.1)		50 (23.0)	7 (25.0)	8 (14.5)	0 (0.0)	1 (14.3)		24 (19.7)	32 (25.8)	10 (15.2)		10 (16.9)	8 (18.6)	48 (22.9)	
A little fear	92 (17.7)	41 (13.1)		33 (15.2)	3 (10.7)	5 (9.1)	0 (0.0)	0 (0.0)		16 (13.1)	18 (14.5)	7 (10.6)		6 (10.2)	3 (7.0)	32 (15.2)	
A fair amount of fear	108 (20.8)	66 (21.2)		50 (23.0)	2 (7.1)	17 (30.9)	2 (40.0)	2 (28.6)		27 (22.1)	32 (25.8)	14 (21.2)		15 (25.4)	14 (32.6)	44 (21.0)	
Much fear	96 (18.5)	73 (23.4)		14 (6.5)	3 (10.7)	4 (7.3)	1 (20.0)	0 (0.0)		8 (6.6)	9 (7.3)	5 (7.6)		3 (5.1)	2 (4.7)	17 (8.1)	
Very much fear	192 (37.0)	110 (35.3)		70 (32.3)	13 (46.4)	21 (38.2)	2 (40.0)	4 (57.1)		47 (38.5)	33 (26.6)	30 (45.5)		25 (42.4)	16 (37.2)	69 (32.9)	
Dying from covid-19 (%)			.805						.341				.663				.002
No fear	71 (13.7)	38 (12.2)		46 (21.2)	5 (17.9)	6 (10.9)	0 (0.0)	2 (28.6)		20 (16.4)	28 (22.6)	11 (16.7)		16 (27.1)	8 (18.6)	35 (16.7)	
A little fear	122 (23.5)	68 (21.8)		50 (23.0)	7 (25.0)	11 (20.0)	0 (0.0)	0 (0.0)		32 (26.2)	25 (20.2)	11 (16.7)		7 (11.9)	7 (16.3)	54 (25.7)	
A fair amount of fear	87 (16.8)	59 (18.9)		36 (16.6)	2 (7.1)	12 (21.8)	2 (40.0)	1 (14.3)		20 (16.4)	21 (16.9)	12 (18.2)		8 (13.6)	11 (25.6)	34 (16.2)	
Much fear	78 (15.0)	53 (17.0)		29 (13.4)	3 (10.7)	5 (9.1)	1 (20.0)	0 (0.0)		14 (11.5)	17 (13.7)	7 (10.6)		2 (3.4)	2 (4.7)	34 (16.2)	
Very much fear	161 (31.0)	94 (30.1)		56 (25.8)	11 (39.3)	21 (38.2)	2 (40.0)	4 (57.1)		36 (29.5)	33 (26.6)	25 (37.9)		26 (44.1)	15 (34.9)	53 (25.2)	
Due to covid-19, my gi condition will get worse (%)			<.001						.105				.169				.183
No fear	175 (33.7)	70 (22.4)		46 (21.2)	6 (21.4)	12 (21.8)	1 (20.0)	0 (0.0)		28 (23.0)	29 (23.4)	8 (12.1)		14 (23.7)	12 (27.9)	39 (18.6)	
A little fear	157 (30.3)	82 (26.3)		62 (28.6)	4 (14.3)	14 (25.5)	1 (20.0)	1 (14.3)		27 (22.1)	37 (29.8)	18 (27.3)		18 (30.5)	13 (30.2)	51 (24.3)	
A fair amount of fear	77 (14.8)	65 (20.8)		33 (15.2)	3 (10.7)	6 (10.9)	0 (0.0)	4 (57.1)		19 (15.6)	16 (12.9)	11 (16.7)		8 (13.6)	9 (20.9)	29 (13.8)	
Much fear	60 (11.6)	46 (14.7)		44 (20.3)	7 (25.0)	17 (30.9)	2 (40.0)	0 (0.0)		30 (24.6)	28 (22.6)	12 (18.2)		9 (15.3)	4 (9.3)	57 (27.1)	
Very much fear	50 (9.6)	49 (15.7)		32 (14.7)	8 (28.6)	6 (10.9)	1 (20.0)	2 (28.6)		18 (14.8)	14 (11.3)	17 (25.8)		10 (16.9)	5 (11.6)	34 (16.2)	
Due to covid-19, there will be less access to the medical support i need for my gi condition (%)			.020						.134				.164				.265
No fear	167 (32.2)	77 (24.7)		40 (18.4)	7 (25.0)	14 (25.5)	0 (0.0)	0 (0.0)		19 (15.6)	27 (21.8)	15 (22.7)		14 (23.7)	11 (25.6)	36 (17.1)	
A little fear	168 (32.4)	89 (28.5)		64 (29.5)	4 (14.3)	15 (27.3)	3 (60.0)	3 (42.9)		33 (27.0)	39 (31.5)	17 (25.8)		20 (33.9)	13 (30.2)	56 (26.7)	
A fair amount of fear	76 (14.6)	61 (19.6)		41 (18.9)	2 (7.1)	12 (21.8)	0 (0.0)	1 (14.3)		24 (19.7)	18 (14.5)	14 (21.2)		8 (13.6)	11 (25.6)	37 (17.6)	
Much fear	65 (12.5)	56 (17.9)		52 (24.0)	10 (35.7)	13 (23.6)	1 (20.0)	1 (14.3)		38 (31.1)	29 (23.4)	10 (15.2)		12 (20.3)	5 (11.6)	60 (28.6)	
Very much fear	43 (8.3)	29 (9.3)		20 (9.2)	5 (17.9)	1 (1.8)	1 (20.0)	2 (28.6)		8 (6.6)	11 (8.9)	10 (15.2)		5 (8.5)	3 (7.0)	21 (10.0)	
Due to covid-19, i’ll be less able to manage my gi condition effectively (%)			.331						.293				.377				.266
No fear	178 (34.3)	89 (28.5)		40 (18.4)	7 (25.0)	10 (18.2)	0 (0.0)	3 (42.9)		21 (17.2)	29 (23.4)	10 (15.2)		13 (22.0)	7 (16.3)	40 (19.0)	
A little fear	173 (33.3)	104 (33.3)		73 (33.6)	7 (25.0)	21 (38.2)	2 (40.0)	1 (14.3)		39 (32.0)	41 (33.1)	24 (36.4)		20 (33.9)	20 (46.5)	64 (30.5)	
A fair amount of fear	77 (14.8)	60 (19.2)		28 (12.9)	5 (17.9)	5 (9.1)	0 (0.0)	1 (14.3)		17 (13.9)	11 (8.9)	11 (16.7)		9 (15.3)	7 (16.3)	23 (11.0)	
Much fear	58 (11.2)	39 (12.5)		62 (28.6)	7 (25.0)	18 (32.7)	2 (40.0)	0 (0.0)		38 (31.1)	37 (29.8)	14 (21.2)		15 (25.4)	6 (14.0)	68 (32.4)	
Very much fear	33 (6.4)	20 (6.4)		14 (6.5)	2 (7.1)	1 (1.8)	1 (20.0)	2 (28.6)		7 (5.7)	6 (4.8)	7 (10.6)		2 (3.4)	3 (7.0)	15 (7.1)	
My gi condition makes me more at risk of getting covid-19 (%)			<.001						.584				.020				.193
No fear	233 (44.9)	50 (16.0)		53 (24.4)	6 (21.4)	13 (23.6)	2 (40.0)	1 (14.3)		30 (24.6)	34 (27.4)	11 (16.7)		9 (15.3)	16 (37.2)	50 (23.8)	
A little fear	159 (30.6)	78 (25.0)		57 (26.3)	5 (17.9)	16 (29.1)	0 (0.0)	0 (0.0)		27 (22.1)	38 (30.6)	13 (19.7)		19 (32.2)	7 (16.3)	52 (24.8)	
A fair amount of fear	64 (12.3)	75 (24.0)		34 (15.7)	4 (14.3)	6 (10.9)	0 (0.0)	3 (42.9)		15 (12.3)	21 (16.9)	11 (16.7)		10 (16.9)	7 (16.3)	30 (14.3)	
Much fear	39 (7.5)	47 (15.1)		34 (15.7)	4 (14.3)	10 (18.2)	1 (20.0)	1 (14.3)		21 (17.2)	19 (15.3)	10 (15.2)		7 (11.9)	4 (9.3)	39 (18.6)	
Very much fear	24 (4.6)	62 (19.9)		39 (18.0)	9 (32.1)	10 (18.2)	2 (40.0)	2 (28.6)		29 (23.8)	12 (9.7)	21 (31.8)		14 (23.7)	9 (20.9)	39 (18.6)	
Having a gi condition means that if i get covid-19 i will be more likely to die (%)			<.001						.045				.023				.725
No fear	227 (43.7)	68 (21.8)		48 (22.1)	9 (32.1)	10 (18.2)	2 (40.0)	0 (0.0)		28 (23.0)	34 (27.4)	7 (10.6)		11 (18.6)	13 (30.2)	45 (21.4)	
A little fear	157 (30.3)	76 (24.4)		57 (26.3)	3 (10.7)	16 (29.1)	0 (0.0)	0 (0.0)		29 (23.8)	35 (28.2)	12 (18.2)		15 (25.4)	8 (18.6)	53 (25.2)	
A fair amount of fear	69 (13.3)	69 (22.1)		26 (12.0)	1 (3.6)	6 (10.9)	1 (20.0)	4 (57.1)		18 (14.8)	11 (8.9)	9 (13.6)		6 (10.2)	7 (16.3)	25 (11.9)	
Much fear	35 (6.7)	38 (12.2)		48 (22.1)	6 (21.4)	12 (21.8)	1 (20.0)	1 (14.3)		27 (22.1)	25 (20.2)	16 (24.2)		12 (20.3)	7 (16.3)	49 (23.3)	
Very much fear	31 (6.0)	61 (19.6)		38 (17.5)	9 (32.1)	11 (20.0)	1 (20.0)	2 (28.6)		20 (16.4)	19 (15.3)	22 (33.3)		15 (25.4)	8 (18.6)	38 (18.1)	
